# Monoaural musical hallucinations caused by a thalamocortical auditory radiation infarct: a case report

**DOI:** 10.1186/1752-1947-8-400

**Published:** 2014-12-02

**Authors:** Peter YM Woo, Lianne NY Leung, Sharon TM Cheng, Kwong-Yau Chan

**Affiliations:** Nursing Quarters, Department of Neurosurgery, Kwong Wah Hospital, Room 318, 25 Waterloo Road, Yaumatei, Hong Kong, SAR China

**Keywords:** Lacunar infarct, Musical hallucination, Stroke, Thalamocortical auditory radiation

## Abstract

**Introduction:**

Musical hallucinations are complex auditory perceptions in the absence of an external acoustic stimulus and are often consistent with previous listening experience. Their causation can be classified as associated with either psychiatric disorders, such as schizophrenia, or organic disorders, such as epilepsy or sensorineural deafness. Non-epileptic musical hallucinosis due to lesions of the central auditory pathway, especially of the thalamocortical auditory radiation, is rare.

**Case presentation:**

We describe the case of an 85-year old ethnic Chinese woman with a history of transient ischemic attacks and chronic bilateral hearing impairment, who experienced an acute onset of left unilateral musical hallucinations. Our patient did not experience any psychiatric symptoms and there was no other neurological deficit. Pure tone audiometry revealed bilateral hypacusis and magnetic resonance imaging revealed a right non-dominant hemisphere sublenticular lacunar infarct of the thalamocortical auditory radiation. Our patient was managed expectantly and after three months her symptoms subsided spontaneously.

**Conclusion:**

We propose that all patients with monoaural musical hallucinations have brain imaging to rule out a central organic cause, especially within the non-dominant hemisphere, regardless of the presence of a hearing impairment.

## Introduction

Musical hallucinations are defined as the abnormal perception of music in the absence of an external acoustic stimulus [1]. The preservation of consciousness is emphasized to make a distinction from complex partial seizure-associated hallucinations. The prevalence of musical hallucinosis is unknown, but it is estimated that less than one percent of general hospital patients and 27% of psychiatric out-patients experience this condition [2, 3]. The etiologies are protean and can be broadly classified as attributed to either a psychiatric disorder or an organic cause. The latter includes otological or neurological causes or a combination of both. Musical hallucinations resulting from focal brain lesions are extremely rare, with fewer than 25 cases reported [4]. We present the case of an elderly woman who experienced transient monoaural musical hallucinosis secondary to a sublenticular lacunar infarct of the auditory thalamocortical radiation.

## Case presentation

**Figure 1:**
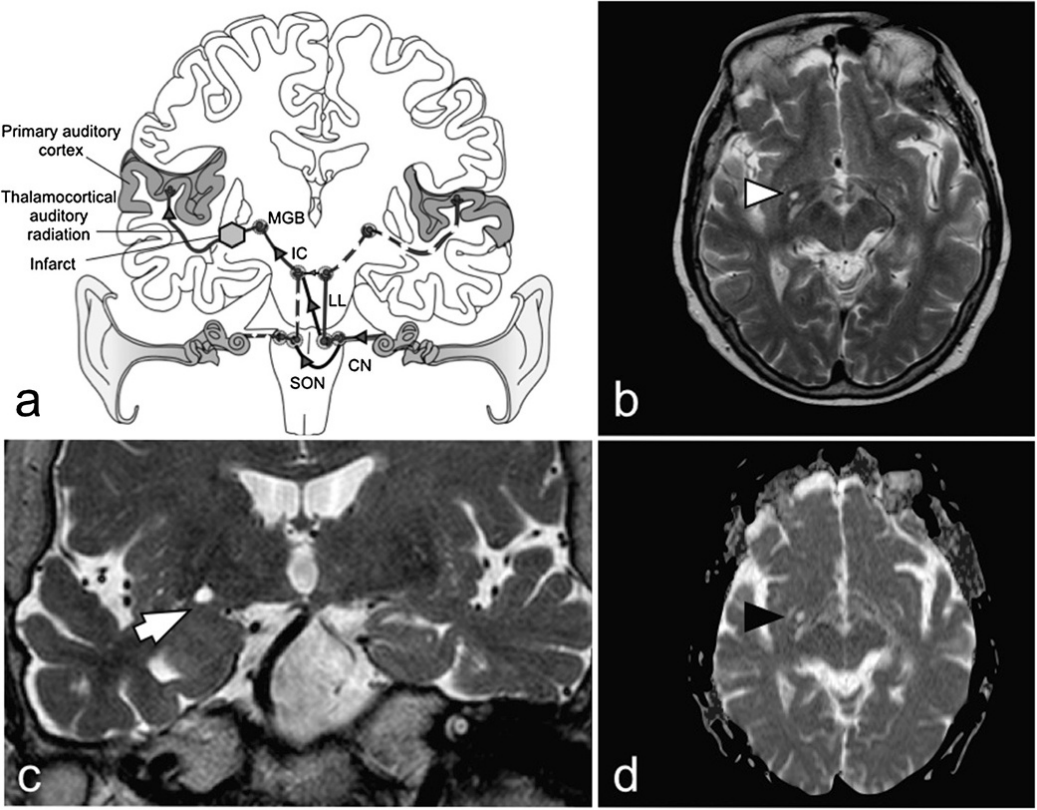
**Central auditory pathway schematic and magnetic resonance imaging features of the patient’s brain. (a)** Schematic of the central auditory pathway. The last subcortical station is the medial geniculate body (MGB), where there is ipsilateral projection of fibers to the primary and association auditory cortices. Extensive bilateral decussations exist from the cochlear nucleus (CN), superior olivary nucleus (SON) and inferior colliculus (IC). The majority of fibers reaching the MGB are derived from the contralateral cochlear nerve (black line with grey arrows; lateral lemniscus (LL)). An infarct of the auditory radiation and sensorineural hearing loss of the contralateral ear could cause contralateral monoaural musical hallucinosis. **(b,c)** T2-weighted magnetic resonance imaging (MRI) sequences showing sublenticular location of infarct (axial, white arrowhead in **b**; coronal, white arrow in **c**). **(d)** Restricted diffusion of the same lesion on diffusion-weighted MRI confirming infarction (black arrowhead).

An 85-year-old bilingual ethnic Chinese woman with chronic bilateral hearing impairment and a history of transient ischemic attacks, characterized by left hemiparesis, experienced sudden intermittent musical hallucinations for three months. The patient was prescribed aspirin due to her history of cerebral ischemia. The hallucinations were perceived exclusively by her left ear and were described as a radio broadcasting of recognizable tunes frequently played during childhood. Vocal and instrumental music were performed in both Chinese, namely in Cantonese and Putonghua dialects, and English. Our patient heard segments of three songs of distinctly different musical genres including the Cantonese opera “The Flower Princess,” a Putonghua folk song “Su Wu, the shepherd,” and an English minstrel song “My Old Kentucky Home.” Initially she was convinced that the music was playing in the room, but soon realized they did not originate from an external source. The hallucinations were intermittent, occurring when the environment was quiet, and discontinued or reduced in volume when she was engaged in conversation. Each episode lasted for five to ten minutes and was not distressing. There were no symptoms indicative of epilepsy or any psychopathology.Psychometric testing revealed our patient to have left hemispheric language dominance. She also had intact general cognition, with only subtle memory impairment as reflected by a Montreal cognitive assessment score of 23 out of 30. Pure tone audiometry confirmed bilateral hypacusis characterized by severe left sensorineural hearing loss, at a threshold of 80dB, and moderate right sensorineural deficit at 45dB. Brainstem auditory evoked potentials were undetectable on her left, but were normal on her right. Electroencephalography did not identify any epileptiform activity. Magnetic resonance imaging (MRI) showed evidence of a right sublenticular lacunar infarct and diffuse cortical atrophy (Figure 1). Our patient was ambivalent to the presence of these hallucinations and refused antipsychotic drug treatment. Aspirin was continued and bilateral hearing aids were prescribed. Three months later her hallucinosis subsided.

## Discussion

Musical hallucinations are complex auditory phenomena pertaining not only to the perception of sound, but also invoke semantic and episodic musical memories [5]. They frequently consist of short fragments of familiar melodies from childhood, including popular songs or religious hymns [4, 6].

Most musical hallucinations are due to psychiatric conditions such as depression, bipolar affective disorder or schizophrenia; non-epileptic organic causes are rare [4]. The exact mechanisms are unclear, but two deafferentation theories have been hypothesized. The peripheral deafferentation theory emphasizes the importance of external sensory stimuli to inhibit the evocation of auditory memories [7–9]. Reminiscent of visual hallucinations in the blind, known as Charles-Bonnet syndrome, it has been postulated that prolonged sensory deprivation inappropriately increases cortical excitability and releases stored memories [7, 8]. Functional neuroimaging suggests that similar distortions of normal sensory information allow abnormal excitation of a distinct musical memory neural substrate [4–6, 10–12]. Single photon emission computed tomography of patients with hearing impairment during active musical hallucinosis revealed spontaneous hyperactivity of the auditory association cortices, including the premotor-motor areas, insula, posterior temporal lobes, basal ganglia and cerebellum [10, 11]. Magnetoencephalography of patients experiencing hallucinations also demonstrated comparable association cortex hyperactivity [11, 12]. Hearing loss was postulated to result in a reduction of the signal-to-noise ratio of incoming auditory stimuli that reinforces a recurrent loop of communication between areas involved in music perception (primary auditory cortex) and music cognition (premotor and motor association cortices) [12]. It was therefore suggested that musical hallucinosis occurs exclusively with acquired hearing loss, especially in older women, and this hypothesis is supported by the observation that hypacusis was the only detectable etiological factor in half of patients observed [4, 13].

In developed countries, a considerable number of individuals, one in seven, have hearing-impairment [4]. The comparatively small number of non-psychiatric patients with musical hallucinations implies that additional central factors, beyond ontological causes may be involved [4, 14]. Disruptions of the musical neural network between the primary and associative cortical auditory centers have been described as a possible mechanism for hallucinogenesis [4, 14]. This central deafferentation theory could accounts for the occasional reports of patients without evidence of otopathy [4, 9, 15, 16].

Focal lesions of the central auditory pathway are rarely described, but shed insight into auditory signal processing and support the central theory for musical hallucinogenesis. Brainstem stroke of the pontine tegmentum, where the cochlear nuclei are located, is the commonest focal lesion observed [9, 16–18]. But sporadic reports of supranuclear lesions of the central auditory pathway situated in the midbrain, thalamus and temporal cortex have been documented [8, 15, 19]. The final subcortical station of the auditory pathway is the thalamic medial geniculate body. This receives ascending neurons via the brachium of the inferior colliculus, the majority of which are derived from the contralateral cochlear nerve (Figure 1) [20]. The auditory thalamocortical radiation subsequently ascends sublenticularly or through the posterior limb of the internal capsule to the primary auditory cortex located at the transverse temporal gyrus, or Heschl’s gyrus, and the association cortices [20].

The findings in our case support the hypothesis that a combination of peripheral, that is, otological, and central deafferentation causes organic musical hallucinosis [16, 19]. We believe that hypacusis with a prolonged lack of normal auditory cortical input might have caused a specific vulnerability, in which subsequent subcortical lacunar infarction of her auditory radiation precipitated the hallucinosis, as reflected by the sudden onset of symptoms. The proximity of the infarct to her superior temporal gyrus and insula may also have disrupted the primary and association cortical musical memory neural network, leading to hyperactivity of the latter [5, 10]. To the best of our knowledge, this is the first reported case of musical hallucinosis secondary to an auditory thalamocortical radiation infarct, although a patient who experienced a putaminal intracerebral hemorrhage in this vicinity was previously reported [15].

Two features of this case are worth noting. First, most patients with organic musical hallucinations secondary to hypacusis experience bilateral symptoms. In our case, we believe the lateralization of symptoms contralateral to the involved hemisphere to be evidence of central deafferentation and cannot be solely attributed to an otological cause. Similar cases of contralateral central lesions have been reported and we propose that monoaural musical hallucinosis is an indication for neuroimaging [14, 18, 21, 22]. Second, this report contributes to accumulating evidence of the importance of hemispheric language dominance and musical training in hallucinosis. Psychometric and functional neuroimaging studies have demonstrated that the non-dominant hemisphere, usually the right side, is responsible for normal music perception in non-musicians [7, 23–25]. Correspondingly, reports, including ours, support the observation that non-dominant right hemispheric lesions may be responsible for hallucinations in the musically untrained [11, 15, 24, 25].

## Conclusion

Our experiences in this case support the proposition that organic musical hallucinogenesis is a result of both peripheral and central deafferentation. To the best of our knowledge, this is the first report of a lacunar infarct of the auditory thalamocortical radiation triggering such symptoms. We recommend that all patients with monoaural musical hallucinations have brain imaging to rule out a central organic cause, regardless of the presence of a hearing impairment.

## Consent

Written informed consent was obtained from the patient for publication of this case report and any accompanying images. A copy of the written consent is available for review by the Editor-in-Chief of this journal.

## Abbreviations

CN: cochlear nucleus
IC: inferior colliculus
LL: lateral lemniscus
MGB: medial geniculate body
MRI: magnetic resonance imaging
SON: superior olivary nucleus.

## References

[1] Simoes S, Mesquita J, Marcal N, Santos M: **Musical hallucinations: case report and review of the literature.** *J Neuropsychiatry Clin Neurosci* 2012,**24**(2)**:**E8. 10.1176/appi.neuropsych.11050107 10.1176/appi.neuropsych.11050107 22772708

[2] Fukunishi I, Horikawa N, Onai H: **Prevalence rate of musical hallucinations in a general hospital setting.** *Psychosomatics* 1998,**39**(2)**:**175. 10.1016/S0033-3182(98)71368-4 10.1016/S0033-3182(98)71368-4 9584545

[3] Hermesh H, Konas S, Shiloh R, Dar R, Marom S, Weizman A, Gross-Isseroff R: **Musical hallucinations: prevalence in psychotic and nonpsychotic outpatients.** *J Clin Psychiatry* 2004,**65**(2)**:**191–197. 10.4088/JCP.v65n0208 10.4088/jcp.v65n0208 15003072

[4] Evers S: **Musical hallucinations.** *Curr Psychiatry Rep* 2006,**8**(3)**:**205–210. 10.1007/s11920-006-0024-0 10.1007/s11920-006-0024-0 19817070

[5] Vanneste S, Song JJ, de Ridder D: **Tinnitus and musical hallucinosis: the same but more.** *Neuroimage* 2013, **82:**373–383. 10.1016/j.neuroimage.2013.05.107 23732881

[6] Vitorovic D, Biller J: **Musical hallucinations and forgotten tunes - case report and brief literature review.** *Front Neurol* 2013, **4:**109. 10.3389/fneur.2013.00109 PMC3737457 23964264

[7] Berrios GE: **Musical hallucinations. A historical and clinical study.** *Br J Psychiatry* 1990, **156:**188–194. 10.1192/bjp.156.2.188 10.1192/bjp.156.2.188 2180526

[8] Isolan GR, Bianchin MM, Bragatti JA, Torres C, Schwartsmann G: **Musical hallucinations following insular glioma resection.** *Neurosurg Focus* 2010,**28**(2)**:**E9. 10.3171/2009.12.FOCUSFOCUS09243 10.3171/2009.12.FOCUSFOCUS09243 20121444

[9] Schielke E, Reuter U, Hoffmann O, Weber JR: **Musical hallucinations with dorsal pontine lesions.** *Neurology* 2000,**55**(3)**:**454–455. 10.1212/WNL.55.3.454 10.1212/wnl.55.3.454 10932294

[10] Griffiths TD: **Musical hallucinosis in acquired deafness. Phenomenology and brain substrate.** *Brain* 2000,**123**(Pt 10)**:**2065–2076. 10.1093/brain/123.10.2065 11004124

[11] Kasai K, Asada T, Yumoto M, Takeya J, Matsuda H: **Evidence for functional abnormality in the right auditory cortex during musical hallucinations.** *Lancet* 1999,**354**(9191)**:**1703–1704. 10.1016/S0140-6736(99)05213-7 10.1016/S0140-6736(99)05213-7 10568580

[12] Kumar S, Sedley W, Barnes GR, Teki S, Friston KJ, Griffiths TD: **A brain basis for musical hallucinations.** *Cortex* 2014, **52:**86–97. 10.1016/j.cortex.2013.12.002 PMC3969291 24445167

[13] Gordon AG: **Do musical hallucinations always arise from the inner ear?** *Med Hypotheses* 1997,**49**(2)**:**111–122. 10.1016/S0306-9877(97)90216-4 10.1016/s0306-9877(97)90216-4 9278923

[14] Bhatt YM, de Carpentier JP: **Musical hallucination following whiplash injury: case report and literature review.** *J Laryngol Otol* 2012,**126**(6)**:**615–618. 10.1017/S0022215112000242 10.1017/S0022215112000242 22643207

[15] Cerrato P, Imperiale D, Giraudo M, Baima C, Grasso M, Lopiano L, Bergamasco B: **Complex musical hallucinosis in a professional musician with a left subcortical haemorrhage.** *J Neurol Neurosurg Psychiatry* 2001,**71**(2)**:**280–281. 10.1136/jnnp.71.2.280 10.1136/jnnp.71.2.280 PMC1737492 11488278

[16] Murata S, Naritomi H, Sawada T: **Musical auditory hallucinations caused by a brainstem lesion.** *Neurology* 1994,**44**(1)**:**156–158. 10.1212/WNL.44.1.156 10.1212/wnl.44.1.156 8290053

[17] Cascino GD, Adams RD: **Brainstem auditory hallucinosis.** *Neurology* 1986,**36**(8)**:**1042–1047. 10.1212/WNL.36.8.1042 10.1212/wnl.36.8.1042 3526177

[18] Dinges M, Riemer T, Schubert T, Pruss H: **Musical hallucinations after pontine ischemia: the auditory Charles Bonnet syndrome?** *J Neurol* 2013,**260**(10)**:**2678–2680. 10.1007/s00415-013-7114-9 10.1007/s00415-013-7114-9 24057151

[19] Inzelberg R, Vishnievskaya S, Korczyn AD: **Transient musical hallucinosis.** *J Neurol Neurosurg Psychiatry* 1993,**56**(7)**:**833. 10.1136/jnnp.56.7.833 PMC1015074 8331367

[20] Hausler R, Levine RA: **Auditory dysfunction in stroke.** *Acta Otolaryngol* 2000,**120**(6)**:**689–703. 10.1080/000164800750000207 10.1080/000164800750000207 11099144

[21] Paquier P, van Vugt P, Bal P, Cras P, Parizel PM, van Haesendonck J, Creten W, Martin JJ: **Transient musical hallucinosis of central origin: a review and clinical study.** *J Neurol Neurosurg Psychiatry* 1992,**55**(11)**:**1069–1073. 10.1136/jnnp.55.11.1069 10.1136/jnnp.55.11.1069 PMC1015294 1469404

[22] Penfield W, Perot P: **The brain’s record of auditory and visual experience. A final summary and discussion.** *Brain* 1963, **86:**595–696. 10.1093/brain/86.4.595 10.1093/brain/86.4.595 14090522

[23] Bever TG, Chiarello RJ: **Cerebral dominance in musicians and nonmusicians. 1974.** *J Neuropsychiatry Clin Neurosci* 2009,**21**(1)**:**94–97. 10.1176/appi.neuropsych.21.1.94 10.1176/jnp.2009.21.1.94 19359458

[24] Cope TE, Baguley DM: **Is musical hallucination an otological phenomenon? A review of the literature.** *Clin Otolaryngol* 2009,**34**(5)**:**423–430. 10.1111/j.1749-4486.2009.02013.x 10.1111/j.1749-4486.2009.02013.x 19793274

[25] Evers S, Ellger T, Ringelstein EB, Knecht S: **Is hemispheric language dominance relevant in musical hallucinations? Two case reports.** *Eur Arch Psychiatry Clin Neurosci* 2002,**252**(6)**:**299–302. 10.1007/s00406-002-0396-z 10.1007/s00406-002-0396-z 12563539

